# Two new species of the interstitial genus *Parvocythere* (Crustacea, Ostracoda, Cytheroidea) from Japan: an example of morphological variation

**DOI:** 10.3897/zookeys.193.2842

**Published:** 2012-05-14

**Authors:** Ryouichi Higashi, Akira Tsukagoshi

**Affiliations:** 1 Department of Geosciences, Faculty of Science, Shizuoka University, Oya 836, Shizuoka City, 422-8529 Japan

**Keywords:** Interstitial animal, Podocopa, taxonomy, antennular suture

## Abstract

Two new species of the interstitial ostracod genus *Parvocythere*, *Parvocythere gottwaldi* **sp. n.** and *Parvocythere gracilis* **sp. n.**, are herein described. Although these two new species are clearly distinguishable by certain morphological differences in elements of the male copulatory organ, and the carapace, they share the following simplified characters of the appendages and male copulatory organ: antennular fourth podomere with no suture; reduced claws on the distal end of antenna; and asymmetric male copulatory organ. The morphological differences among known and new *Parvocythere* species suggest that the species of this genus can be classified into two groups by the presence/absence of the suture on the antennular fourth podomere. The “Group S” is characterised by the presence of the antennular suture, and all species of this group have a two-clawed antenna and symmetric male copulatory organ, characters which are generally seen in cytheroid ostracods. The species belonging to “Group N” are characterised by the absence of the suture, regarded as a pedomorphic character, show the following characters: two clawed or one clawed antenna, and symmetric or asymmetric male copulatory organ. The morphological variation within Group N includes reductive characters regarded as an adaptation to the narrow spaces of the interstitial environment of a sandy beach. These intrageneric morphological variations of the exclusively interstitial genus *Parvocythere* suggest the possibilities that Group N might be derived from Group S, and that some adaptive characters to an interstitial environment could have developed after the colonisation of these environments.

## Introduction

The genus *Parvocythere* Hartmann, 1959 includes 14 species (type species: *Parvocythere dentata* Hartmann, 1959), all of which are known as interstitial dwellers, inhabiting sedimentary interstices. This genus is characterised by a small body size, only two pairs of walking legs (Hartmann 1959, Marinov 1962), antennula consisting of short podomeres (Hartmann 1974) and (extremely) reduced eyes (Gottwald 1983). These diagnostic characters are regarded as a result of adaptation to the interstitial environments with no light and narrow space (Hartmann 1959, 1973). The genus *Parvocythere* is a suitable taxon for understanding the morphological evolution of interstitial ostracods.

Gottwald (1983) classified the *Parvocythere* species into the following two groups based on the morphology of the male copulatory organ: the *elongata* group characterised by four sclerotised frameworks circularly connected at the base of the capsule, short distal lobe and a short copulatory duct, and the *dentata* group characterised by the lack of circularly connected frameworks, bi- or tri-forked distal lobe and a strongly sclerotised long copulatory duct. This division, however, is controversial in view of the evolutionary clusters, because many morphological characters of the carapace and appendages are not shared within each group. Moreover, since it is known that the characters of male copulatory organ are frequently restricted by character displacement rather than phylogeny in Ostracoda (Tsukagoshi 1988), grouping based on male copulatory organ morphology such as the *elongata* and *dentata* groups should be done carefully.

*Parvocythere japonica* Watanabe, Tsukagoshi & Higashi, 2008 was so far known as the single species belonging to this genus in Japan. In the present paper, two new *Parvocythere* species from central Japan are described, and the evolutionary trend of the genus *Parvocythere* is discussed on the basis of the interspecific morphological variations among the *Parvocythere* species.

## Materials and methods

**Figure 1.**
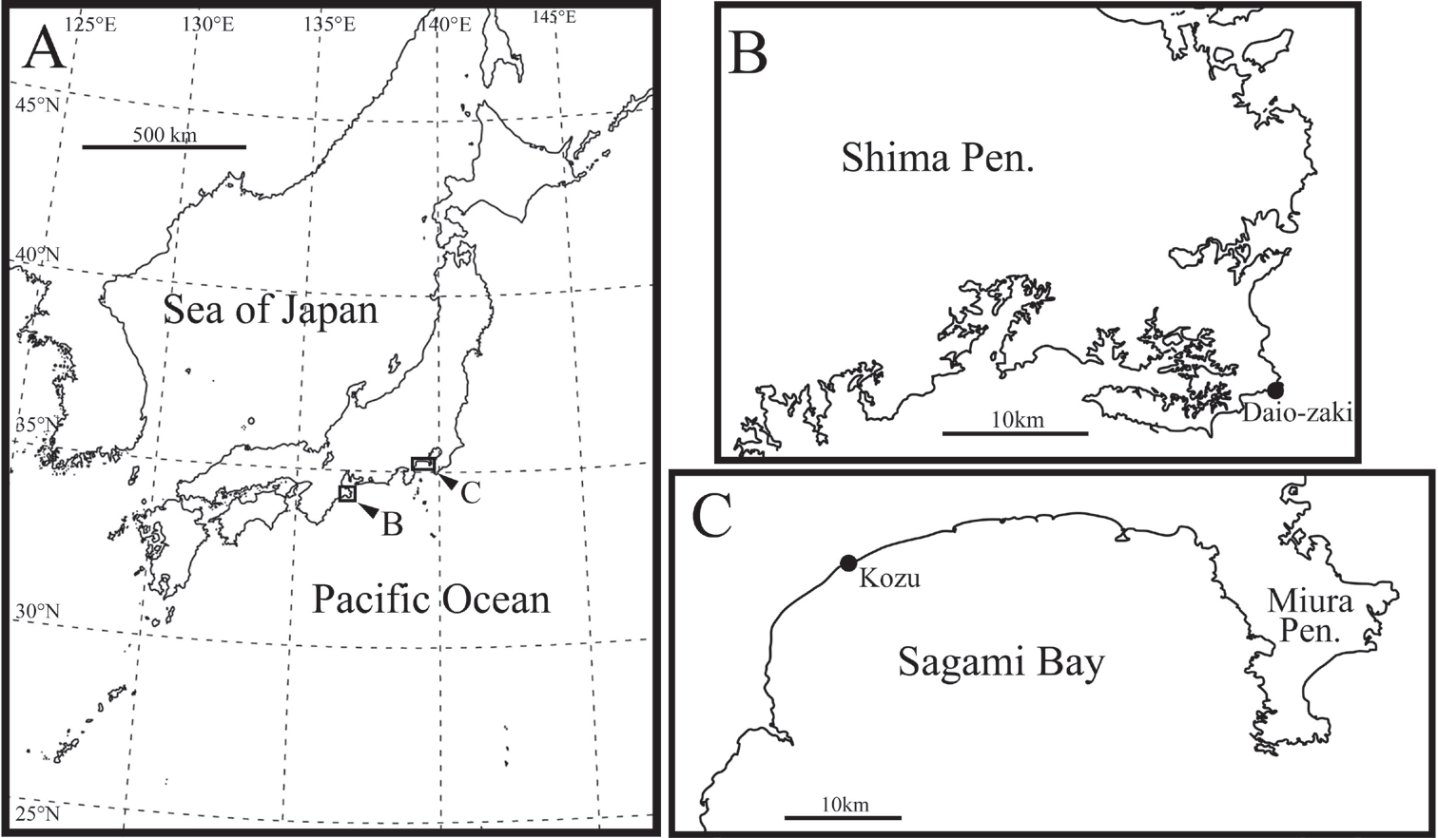
Sampling localities. **A** map of Japan **B** and **C** sampling sites.

Sediment samples were collected from littoral beaches at two sites in central Japan (Fig. 1): Daio-zaki, Shima City, Mie Prefecture (34°16.59'N, 136°53.83'E), and Kozu, Odawara City, Kanagawa Prefecture (35°16.73'N, 139°12.75'E). The sediments were taken with a small scoop from the bottom of a hole dug with a shovel to depth of water table. The samples were washed five times in a bucket of fresh water in the laboratory, and the supernatant was then strained through a 25 µm mesh sieve. The specimens of *Parvocythere* were then picked out under a binocular microscope (SHZ-10, OLYMPUS), from the concentrate retained on the sieve.

The specimens were dissected under a binocular microscope, chitinous parts mounted on glass slides and the valves air-dried. The chitinous parts and the valves were observed and drawn using a differential interference contrast microscope with a camera lucida (BX 50, Olympus). The valves were osmium-coated with an osmium plasma coater (OPC 40, Nippon Laser), and were then observed under a SEM (JSM-5600LV, JEOL).

The type series is deposited in the collection of the Shizuoka University Museum, identified by a number with the prefix SUM-CO.

## Taxonomy

### Order Podocopida Sars, 1866. Superfamily Cytheroidea Baird, 1850. Family Parvocytheridae Hartmann, 1959. Genus Parvocythere Hartmann, 1959

#### Parvocythere gottwaldi sp. n.

urn:lsid:zoobank.org:act:DA15A671-D4D4-427C-A92D-3AC27CE729D7

http://species-id.net/wiki/Parvocythere_gottwaldi

**Figure 2.**
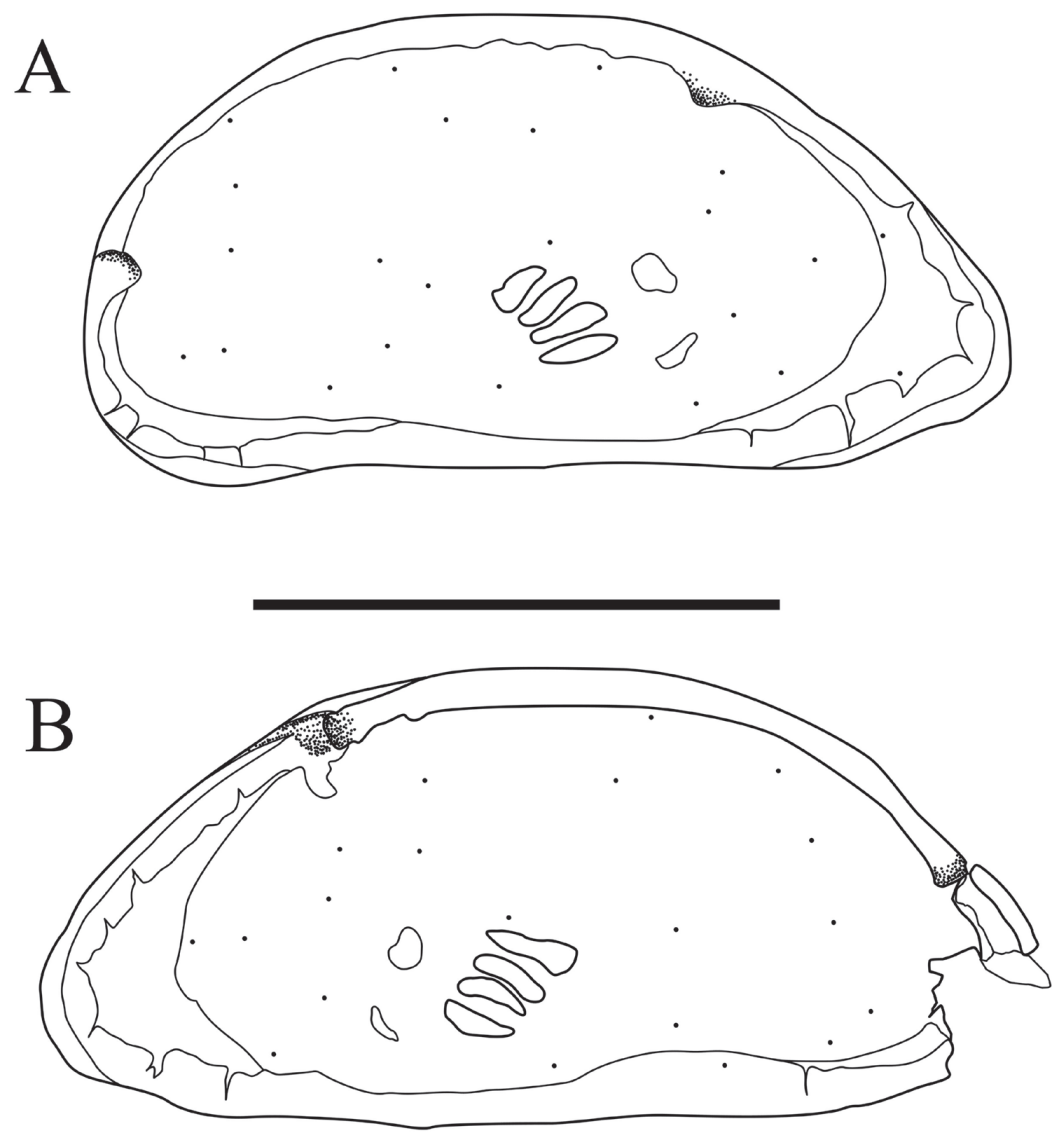
Carapaces of *Parvocythere gottwaldi* sp. n. Holotype (SUM-CO-2023). **A** right external view **B** left external view. Each of the carapace structures are transmitted images. Scale bar indicates 100 µm.

Figs 2 [Fig F3] [Fig F4] [Fig F5] –6

##### Type series.

Holotype: adult male (SUM-CO-2023), right valve length 173 µm, height 88 µm, left valve length 174 µm, height 84 µm, appendages mounted on slide and valves preserved in a cardboard cell slide, Paratypes: 14 adult males (SUM-CO-2024–2037) and 12 adult females (SUM-CO-2038–2049). All illustrated specimens were collected from interstitial pore-water at the type locality on October 10, 2008.

##### Type locality.

 Daio-zaki, Shima City, Mie Prefecture, Pacific coast of central Japan, 34°16.59'N, 136°53.83'E (Fig. 1B), in sediments at approximately 20 cm depth. The sediment is mainly composed of clastic very coarse sand and granules.

##### Etymology.

This species is named in honour of Dr Jochen Gottwald, in recognition of his significant contribution to our knowledge of interstitial ostracods.

##### Diagnosis.

 Carapace rounded trapezial in lateral view with very slight protrusion on antero-ventral area and sharp wrinkle-like groove running from middle to posterior in ventral area of both valves. Tapering anterior margin. Forty-two pore systems per valve. Seven and four marginal pores along anterior and posterior margins, respectively. Marginal infold broad in anterior but very narrow in posterior. Hingement modified pentodont type. No suture on middle of fourth podomere of antennula. Antenna with only one distal claw. Male copulatory organ asymmetric, with left organ (hemipenis) larger than right one. Left hemipenis bearing long copulatory duct single coiled in distal part, short and stout claw-like dorsal ramus, two-pronged ventral ramus, and well-developed crescent-shaped distal lobe with thick seta on centre of proximal part. Right organ reduced and bearing much reduced copulatory duct and crescent-shaped distal lobe with thick seta on proximal part.

##### Description.

**Figure 3. F3:**
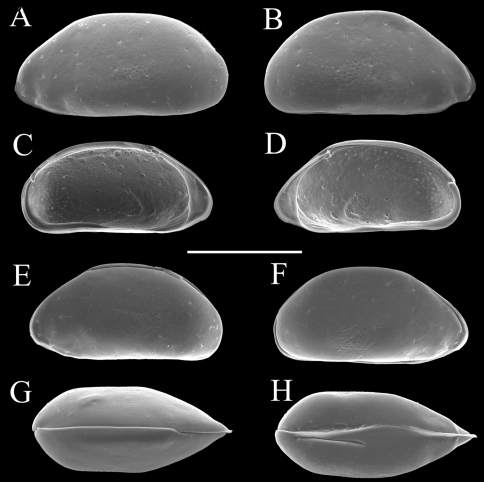
Carapaces of *Parvocythere gottwaldi* sp. n. **A–D**, **G** and **H** male specimens: **A** and **B** paratype (SUM-CO-2025) **C** and **D** paratype (SUM-CO-2026) **G** paratype (SUM-CO-2027) **H** paratype (SUM-CO-2028). **A** left external lateral view **B** right external lateral view **C** internal view of left valve **D** internal view of right valve **G** dorsal view **H** ventral view. **E** and **F** female specimens: **E** paratype (SUM-CO-2039) **F** paratype (SUM-CO-2040). **E** left external view **F** right external view. Scale bar indicates 100 μm.

 Carapace (Figs 2 and 3). Carapace rounded trapezial in lateral view with very slight protrusion on antero-ventral area and sharp wrinkle-like groove running from middle to posterior in ventral area of both valves. Anterior margin tapers more than posterior margin. Dorsal margin slightly rounded. Ventral margin almost straight. Carapace surface generally smooth. All pore systems of simple type and 42 per valve. Seven and four marginal pores along anterior and posterior margins, respectively. Marginal infold broad in anterior area but very narrow in posterior area. Vestibula occupying large part of marginal infold. Inner surface covered with numerous pits. Hingement modified pentodont type. Right valve slightly overlapping left valve along hinge line. Four adductor muscle scars in oblique row. Mandibular scar visible beneath frontal scar.

**Figure 4. F4:**
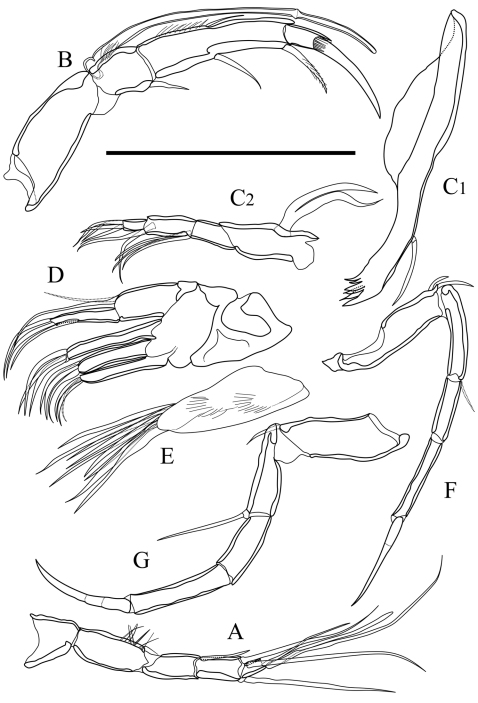
Appendages of *Parvocythere gottwaldi* sp. n. **A–D**, **F** and **G** holotype (SUM-CO-2023) **E** paratype (SUM-CO-2024). **A** antennula **B** antenna **C_1_** coxa of mandibula **C_2_** palp of mandibula **D** palp and endites of maxillula **E** branchial plate of maxillula **F** fifth limb **G** sixth limb. Scale bar indicates 50 µm.

Antennula (Fig. 4A). Five articulated podomeres. First podomere bare and short. Second podomere nine quarters as long as first podomere, with fine setae along antero-distal margin. Third podomere half as long as second podomere, with one medium seta on antero-distal end. Fourth podomere as long as third podomere, with two long setae on antero-distal end and one long seta on postero-distal end. Fifth podomere half as long as fourth podomere, with one long and one very long simple setae and one spatula-like long seta on distal end.

Antenna (Fig. 4B). Four articulated podomeres. First podomere with two segmented spinneret (exopodite) on distal end. Second podomere half as long as first podomere, with bunch of fine setae along middle of anterior margin and one medium seta on postero-distal end. Third podomere eight-thirds as long as second podomere, with numerous fine setae along antero-proximal margin, medium setae on middle of anterior margin and on middle of posterior margin, respectively, and one medium setulous seta on postero-distal end. Fifth podomere quarter length of fourth podomere, with numerous fine setae on distal margin and one stout claw on distal end.

Mandibula (Fig. 4C_1_, C_2_). Coxa (Fig. 4C_1_) elongated, with one medium seta on antero-ventral part. Six coxal endites. Palp (Fig. 4C_2_) consisting of four articulated podomeres. First podomere (basis) with bifurcated lamella (exopodite) on middle of dorsal margin. Second podomere two-thirds as long as first podomere, with three medium setae on ventro-distal end. Third podomere five-fourths as long as second podomere, with one stout medium seta on outside of distal end and one medium seta on ventro-distal end. Fourth podomere two-fifths as long as third podomere, with four setae on distal end.

Maxillula (Fig. 4D and E). Thin branchial plate (exopodite; Fig. 4E) with approximately nine plumose setae. Basal podomere with one palp and three endites (Fig. 4D). Palp consisting of two articulated podomeres: first podomere with one long and one medium seta on antero-distal end; second podomere eleven-tenth as long as first podomere, with one medium seta on middle of posterior margin and two medium setae on distal end. Endites with three setae, respectively.

Fifth limb (Fig. 4F). Four articulated podomeres. First podomere with two short setae on antero-distal end. Second podomere five-sixths as long as first podomere, with one medium seta on antero-distal end. Third podomere four-fifths as long as second podomere. Fourth podomere three-halves as long as third podomere, with stout distal claw.

Sixth limb (Fig. 4G). Four articulated podomeres. First podomere with one short seta on antero-distal end. Second podomere four-fifth as long as first podomere, with one long seta on antero-distal end. Third podomere as long as second podomere. Fourth podomere seven-fifth as long as third podomere with stout distal claw.

Seventh limb. Absent.

**Figure 5. F5:**
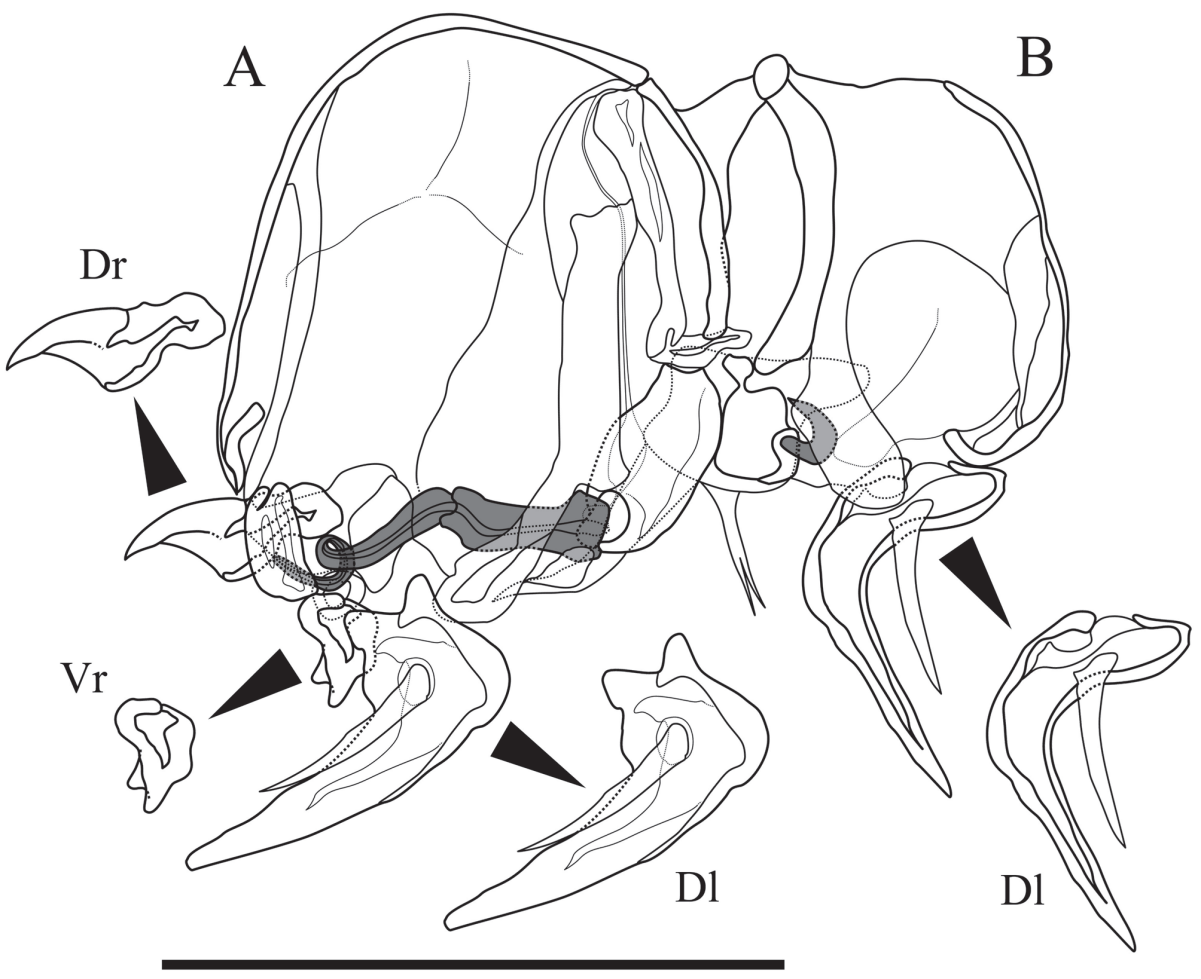
Male copulatory organs of *Parvocythere gottwaldi* sp. n. Holotype (SUM-CO-2023). **A** external view of left organ **B** external view of right organ. Copulatory ducts are shaded. Abbreviation: **Dr** dorsal ramus **Vr** ventral ramus **Dl** Distal lobe. Scale bar indicates 50 µm.

Male copulatory organ (Fig. 5). Asymmetric. Right organ (Fig. 5B) smaller than left (Fig. 5A), with reduced components: square capsule with weakened framework; copulatory duct (Cd) extremely reduced; distal lobe (Dl) thin and crescent-shaped with thick seta on proximal part. Left organ bearing well-developed long copulatory duct (Cd) single coiled in distal part; short and stout claw-like dorsal ramus (Dr); two-pronged ventral ramus (Vr); and crescent-shaped distal lobe (Dl) with thick seta on centre of proximal part.

**Figure 6.**
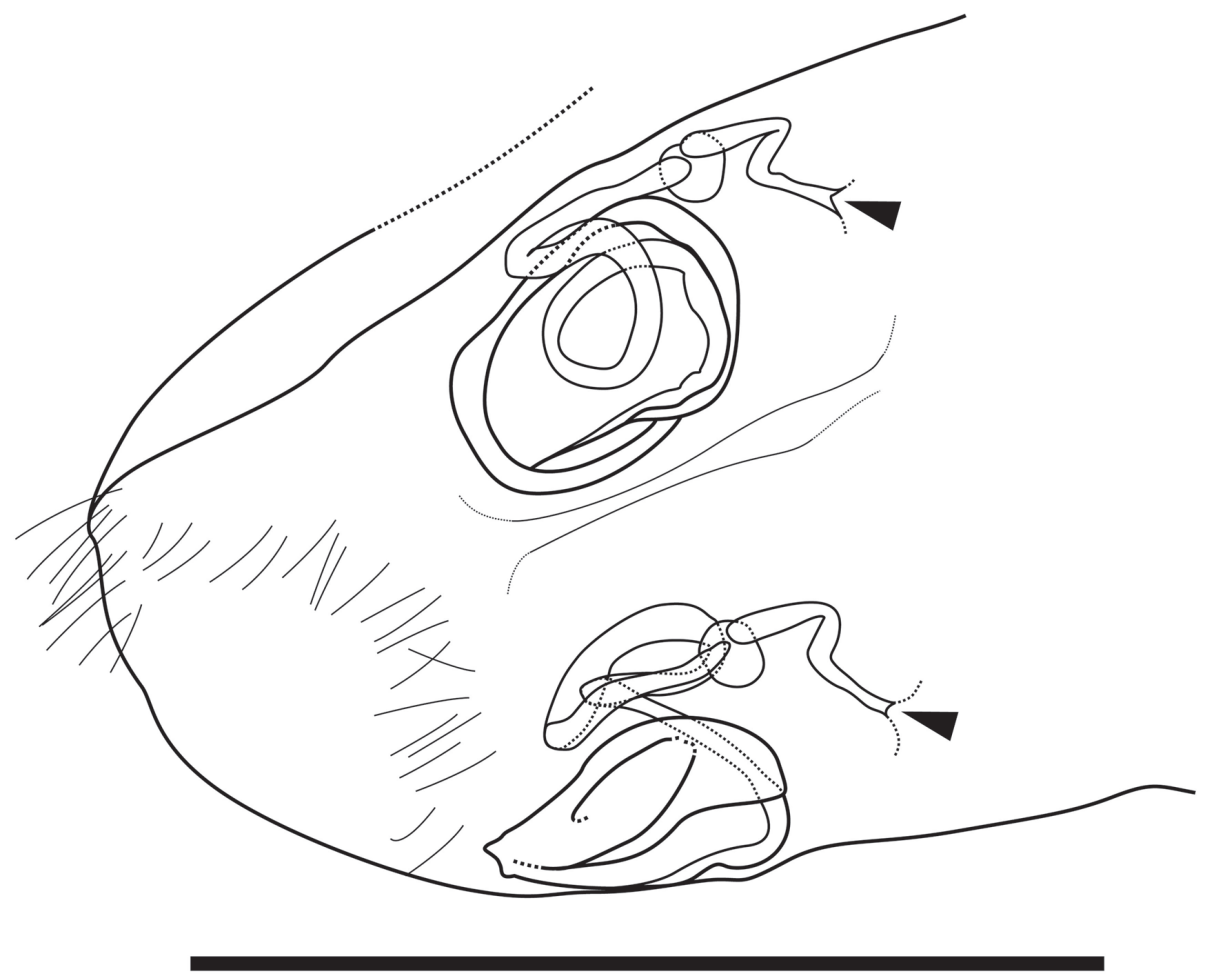
Caudal part of female of *Parvocythere gottwaldi* sp. n. Dorsal view (paratype, SUM-CO-2038). Arrows indicate openings. Scale bar indicates 50 µm.

Genitalia of female (Fig. 6). Almost symmetric and consisting of rounded frame-work and winding duct with vesicle-like joint nearby opening. Duct opening on ventral side.

Eye. Absent.

##### Dimensions.

 See Table 1.

**Table 1.** Dimensions of valves of *Parvocythere gottwaldi* sp. n. from type locality.<br/>

		**Length (µm)**			**Height (µm)**		
		**Mean**	**Observed range**	**N**	**Mean**	**Observed range**	**N**
Male	Right valve	170	165–175	11	87	85–92	11
	Left valve	173	169–176	11	85	82–87	11
Female	Right valve	167	164–174	9	88	84–95	9
	Left valve	171	168–176	7	86	83–91	7

##### Occurrences.

Type locality and Kozu, Odawara City, Kanagawa Prefecture, Pacific coast of central Japan (35°16.73'N, 139°12.75'E).

##### Remarks.

*Parvocythere gottwaldi* sp. n. resembles *Parvocythere mauiensis* Hartmann, 1991 in the outline of carapace. The new species, however, can be distinguished from the other species by its antenna with only one distal claw and the asymmetric male copulatory organ. Although this new species is also similar to *Parvocythere* spec. A, as referred to in Gottwald (1983), in the characteristics of antenna and asymmetric male copulatory organ, the new species can be distinguished from *Parvocythere* spec. A by the tapering anterior margin of carapace and the copulatory duct (Cd) single coiled in the distal part.

#### Parvocythere gracilis sp. n.

urn:lsid:zoobank.org:act:8F4E53A3-FACF-48D2-8862-CC4634E2503D

http://species-id.net/wiki/Parvocythere_gracilis

**Figure 7.**
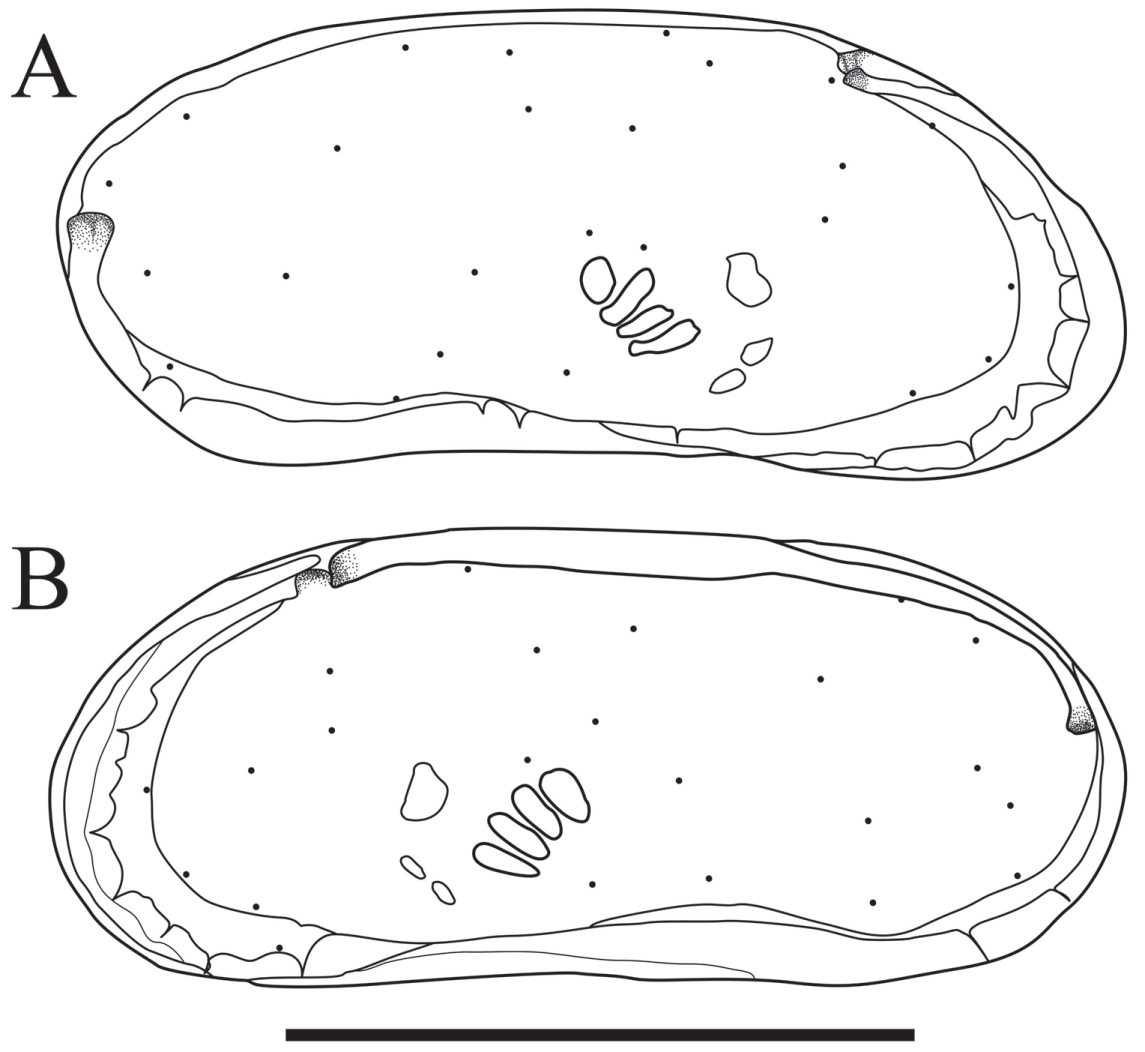
Carapaces of *Parvocythere gracilis* sp. n. Holotype (SUM-CO-2050). **A** right external view **B** left external view. The carapace structures are transmitted images. Scale bar indicates 100 µm.

Figs 7 [Fig F8] [Fig F9] [Fig F10] –11

##### Type series.

Holotype: adult male (SUM-CO-2050), right valve length 170 µm, height 75 µm, left valve length 169 µm, height 73 µm, appendages mounted on slide and valves preserved in a cardboard cell slide, Paratypes: 7 adult males (SUM-CO-2051–2057) and 8 adult females (SUM-CO-2058–2065). All illustrated specimens were collected from interstitial pore-water at the type locality on April 16, 2010.

##### Type locality.

 Kozu, Odawara City, Kanagawa Prefecture, Pacific coast of central Japan, 35°16.73'N, 139°12.75'E in sediments at approximately 20 cm depth. The sediment is mainly composed of clastic granules and pebbles.

##### Etymology.

The Latin *gracilis* (slender) refers to the slender aspect of the carapace of this species.

##### Diagnosis.

 Carapace elongated and bean-shaped in lateral view. Anterior margin slightly extending like thin plate in ventral and dorsal views. Middle of posterior margin of right valve slightly pointed in lateral view. Forty-two pore systems per valve. Six and two marginal pores along anterior and posterior margins, respectively. Marginal infold narrow in anterior and ventral, and very narrow in posterior. Hingement modified pentodont type. No suture on middle of fourth podomere of antennula. Antenna with only one stout claw and one tiny seta on distal end. Male copulatory organ asymmetry: left organ larger than right one. Left organ bearing long L-shaped copulatory duct, dorsal ramus with two small projections, blunt two-pronged ventral ramus, and well-developed crescent-shaped distal lobe with one conspicuous seta on its centre; right organ reduced and bearing extremely reduced copulatory duct and well-developed crescent-shaped distal lobe with thick seta on proximal part.

##### Description.

**Figure 8. F8:**
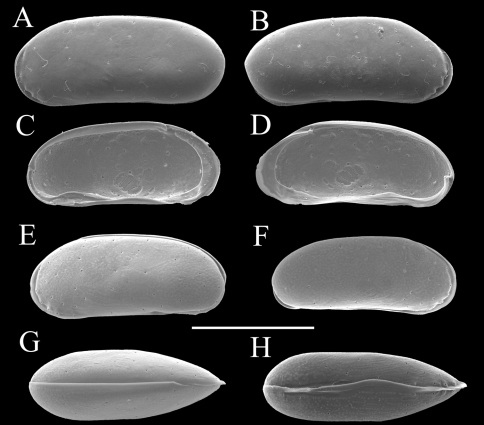
Carapaces of *Parvocythere gracilis* sp. n. **A–D** and **H** male specimens: **A** paratype (SUM-CO-2051) **B** paratype (SUM-CO-2052) **C** and **D** paratype (SUM-CO-2053) **H** paratype (SUM-CO-2054). **A** left external lateral view **B** right external lateral view **C** internal view of left valve **D** internal view of right valve **H** ventral view. **E–G** female specimens: **E** paratype (SUM-CO-2059) **F** paratype (SUM-CO-2059) **G** paratype (SUM-CO-2060). **E** left external view **F** right external view **G** dorsal view. Scale bar indicates 100 μm.

 Carapace (Figs 7, 8). Carapace elongated and bean-shaped in lateral view. Anterior margin gently rounded in lateral view. Anterior part slightly extending like thin plate in ventral and dorsal views. Posterior margin gently rounded in left valve and slightly pointed at middle height of right valve. Dorsal margin slightly rounded. Ventral margin almost straight. Carapace surface smooth. All pore-systems of simple type and 42 per valve. Six and two marginal pores along anterior and posterior margins, respectively. Marginal infold narrow in anterior and ventral, and very narrow in posterior area. Vestibula occupying large part of marginal infold. Inner surface covered with numerous pits. Hingement modified pentodont type. Right valve slightly overlapping left valve along hinge line. Four adductor muscle scars in oblique row. Two separated mandibular scars visible beneath frontal scar.

**Figure 9. F9:**
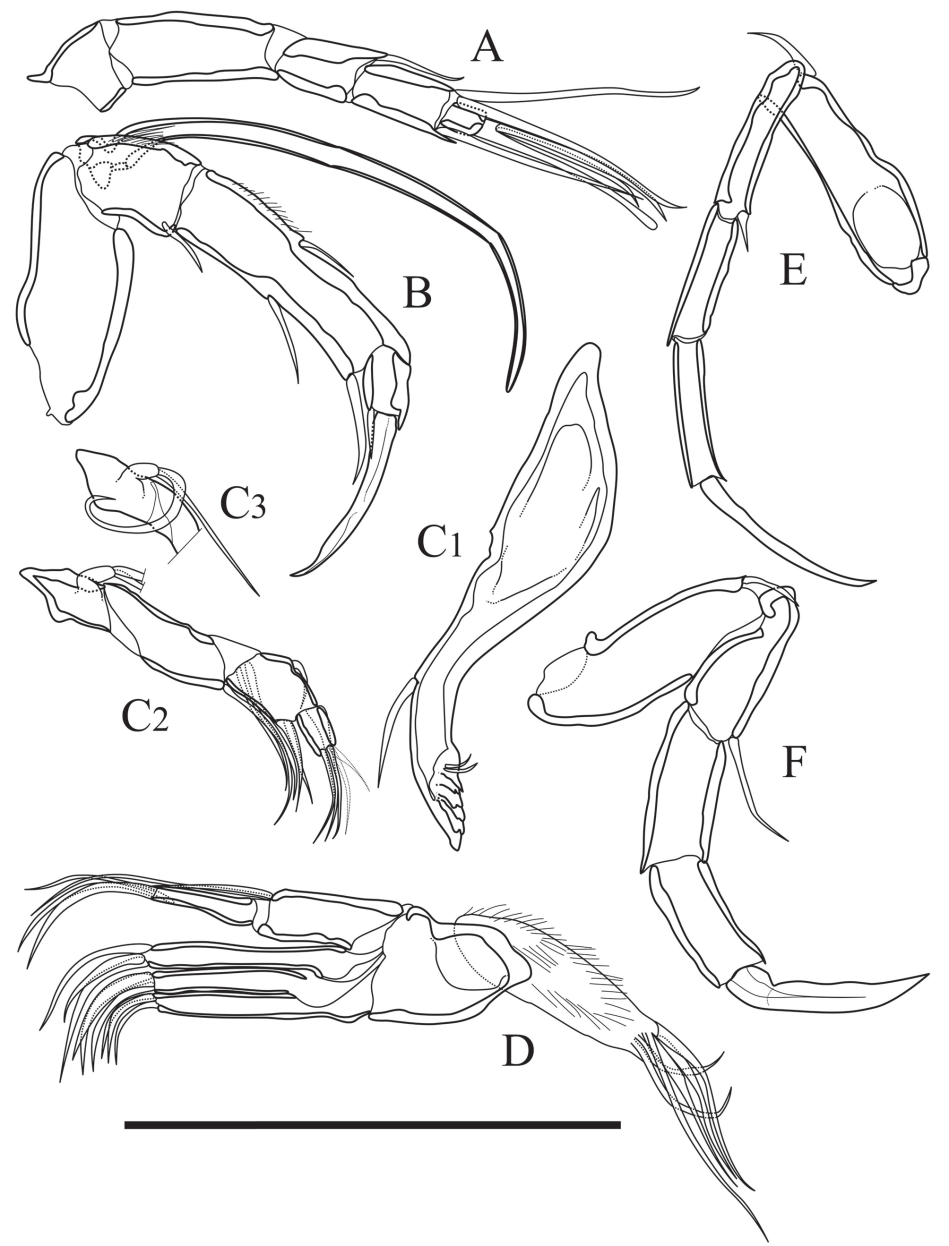
Appendages of *Parvocythere gracilis* sp. n. Holotype (SUM-CO-2050). **A** antennula **B** antenna **C_1_** coxa of mandibula **C_2_** palp of mandibula **C_3_** proximal part of mandibular palp **D** maxillula **E** fifth limb **F** sixth limb. Scale bar indicates 50 µm.

Antennula (Fig. 9A). Five articulated podomeres. First podomere bare and short. Second podomere three times as long as first podomere and bare. Third podomere half as long as second podomere, with one medium seta on antero-distal end. Fourth podomere eleven-tenths as long as third podomere, with two very long setae on antero-distal end and one long seta on postero-distal end. Fifth podomere three-eighths as long as fourth podomere, with two long and one long spatula-like setae on distal end.

Antenna (Fig. 9B). Four articulated podomeres. First podomere with indistinct two-segmented spinneret (exopodite) on distal end. Second podomere half as long as first podomere, with bunch of fine setae at middle of anterior margin and one short seta on postero-distal end. Third podomere five-halves as long as second podomere, with numerous fine setae along antero-proximal margin, one short seta on middle of anterior margin, one medium seta on middle of posterior margin, and one short and thick seta on postero-distal end. Fifth podomere two-seventh as long as fourth podomere, with one stout claw and one very short seta on distal end.

Mandibula (Fig. 9C_1_, C_2_, C_3_). Coxa (Fig. 9C_1_) elongated, with one seta on antero-ventral part, two very short setae on postero-ventral part, and six coxal endites. Palp (Fig. 9C_2_) consisting of four articulated podomere. First podomere (basis) with bifurcated lamella (exopodite; Fig. 9C_3_) on middle of dorsal margin. Second podomere twice as long as first podomere, with three long setae on outside of distal margin. Third podomere two-thirds as long as second podomere, with one short seta on middle of dorsal margin, one medium seta on outside of distal end and two short setae on ventro-distal end. Fourth podomere four-sevenths as long as third podomere, with one short and three medium setae on distal end.

Maxillula (Fig. 9D). Thin branchial plate (exopodite) with approximately six plumose setae. Basal podomere with one palp and three endites. Palp consisting of two articulated podomeres: first podomere with one long and one medium setae on antero-distal end; second podomere as long as first podomere, with one medium seta on middle of posterior margin and two medium setae on distal end. Endites with three setae at the distal end.

Fifth limb (Fig. 9E). Four articulated podomeres. First podomere with one short seta on antero-distal end. Second podomere four-thirds as long as first podomere, with one very short seta on antero-distal end. Third podomere nine-tenths as long as second podomere. Fourth podomere eleven-tenths as long as third podomere, with long distal claw.

Sixth limb (Fig. 9F). Four articulated podomeres. First podomere with one short seta on antero-distal end. Second podomere two-thirds as long as first podomere, with one medium seta on antero-distal end. Third podomere nine-tenths as long as second podomere. Fourth podomere as long as third podomere with well-developed stout distal claw.

Seventh limb. Absent.

**Figure 10. F10:**
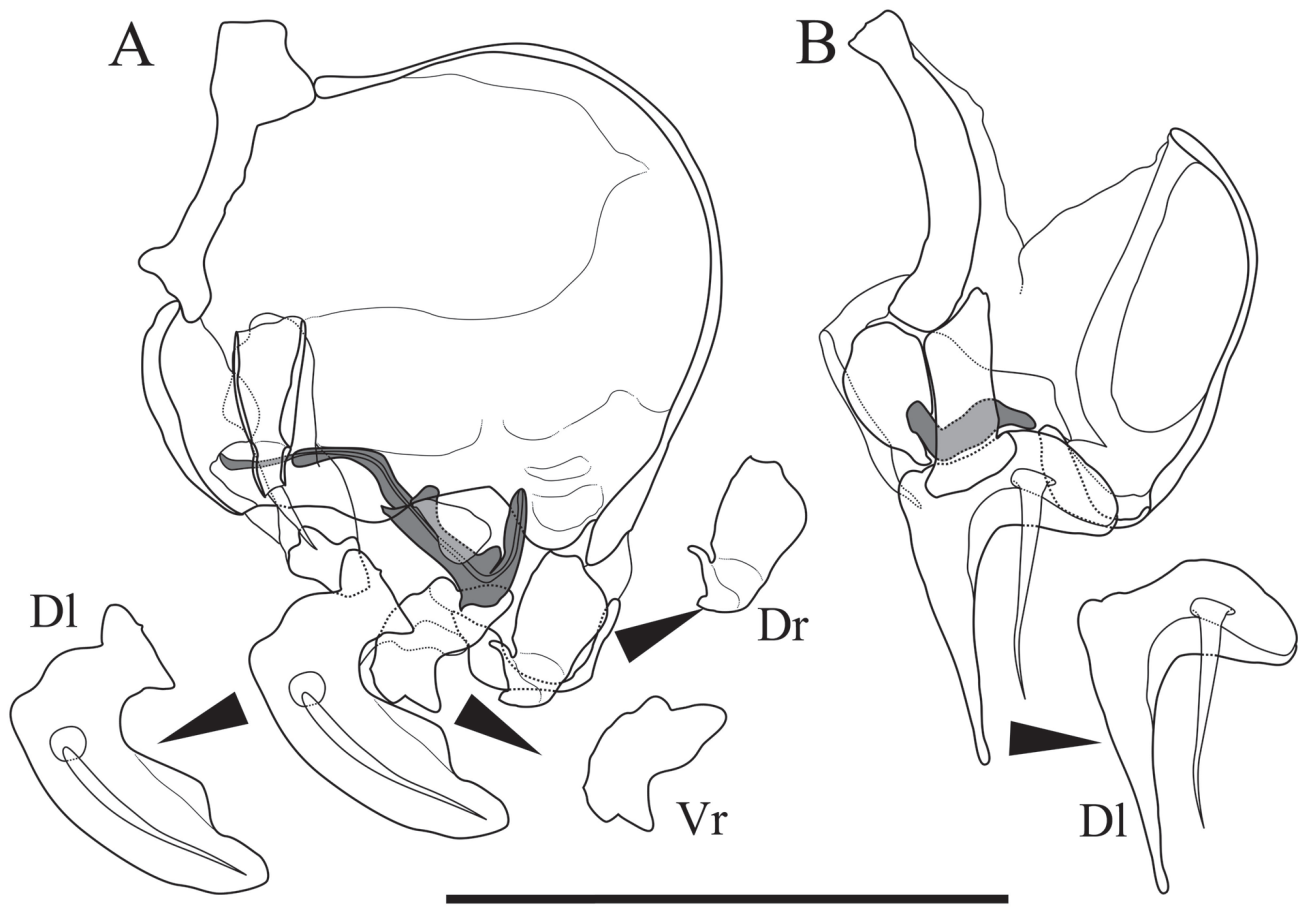
Male copulatory organs of *Parvocythere gracilis* sp. n. Holotype (SUM-CO-2050). **A** internal view of left organ **B** external view of right organ. Copulatory ducts are shaded. Abbreviation: **Dr** dorsal ramus **Vr** ventral ramus **Dl** Distal lobe. Scale bar indicates 50 µm.

Male copulatory organ (Fig. 10). Asymmetric. Right organ smaller than left, with reduced components: square capsule with weakened framework; copulatory duct (Cd) extremely reduced; and distal lobe (Dl) thin and crescent-shaped with thick seta on proximal part. Left male copulatory organ bearing L-shaped long copulatory duct (Cd), dorsal ramus (Dr) with two projections, two-pronged blunt ventral ramus (Vr), and crescent-shaped and well-developed blunt-tipped distal lobe (Dl) with thick seta on centre.

**Figure 11.**
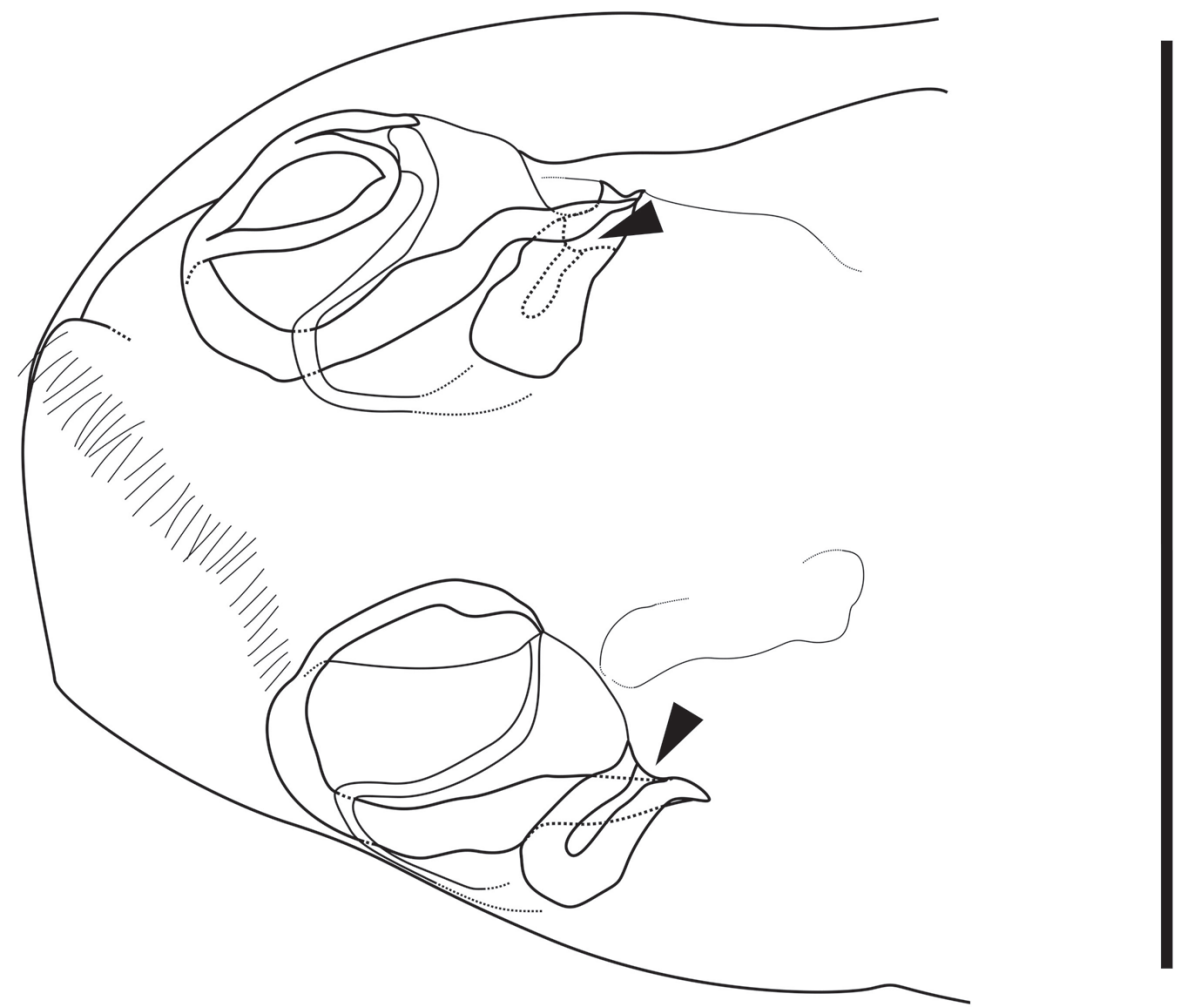
Caudal part of female of *Parvocythere gracilis* sp. n. Dorsal view (paratype, SUM-CO-2058). Arrows indicate openings. Scale bar indicates 50 µm.

Genitalia of female (Fig. 11). Symmetric and consisting of rounded frame-work, sigmoid duct connected with strongly sclerotised opening. Duct opening on ventral side.

Eye. Absent.

##### Dimensions.

 See Table 2.

**Table 2.** Dimensions of valves of *Parvocythere gracilis* sp. n. from type locality.<br/>

		**Length (µm)**			**Height (µm)**		
		**Mean**	**Observed range**	**N**	**Mean**	**Observed range**	**N**
Male	Right valve	166	163–170	4	73	71–75	4
	Left valve	164	162–169	5	71	70–73	5
Female	Right valve	158	156–159	4	70	69–71	4
	Left valve	154	152–157	6	68	67–68	6

##### Occurrences.

Type locality and Daio-zaki, Shima City, Mie Prefecture, Pacific coast of central Japan, (34°16.59'N, 136°53.83'E).

##### Remarks.

*Parvocythere gracilis* sp. n. resembles *Parvocythere galapagoensis* Gottwald, 1983, *Parvocythere schmidti* Gottwald, 1983 and *Parvocythere subterranea* Gottwald, 1983 in the shape of carapace. The new species, however, is slenderer than the other species, and can be distinguished from them by the following reduced characters: no rib posterior to adductor muscle scars; no suture on middle of antennular fourth podomere; only one developed distal claw of antenna; and asymmetric male copulatory organ. Although *Parvocythere gracilis* sp. n. also resembles *Parvocythere elongata* Hartmann, 1959 and *Parvocythere supralitoralis* Gottwald, 1983 in the shape of carapace and the reductive characters of antennula and antenna, only the new species has asymmetric male copulatory organ. Moreover, *Parvocythere gracilis* sp. n. is also similar to *Parvocythere dimorpha* Hartmann, 1974 in the shape of the carapace, the many characters of appendages and an asymmetric male copulatory organ but the new species differs from other species in the number of antennal distal claw, the thickness of sixth limb and the form of copulatory duct.

## Discussion

The *Parvocythere* species, including the new species *Parvocythere gottwaldi* sp. n. and *Parvocythere gracilis* sp. n., are remarkably varied in the characters of antennula, antenna and male copulatory organ (Table 3).

**Table 3.** Character states of *Parvocythere* species. Asterisked species have remarkably small length (<160 µm). Abbreviations: S, Group S; N, Group N; 1+1, one claw and one seta on distal end of antenna; sym, symmetric; asym, asymmetric; D, *dentata* group; E, *elongata* group.<br/>

**Species**	**Body length (µm)**	**Suture on antennular 4th podomere**	**Grouping in this study**	**Number of antennal distal claw**	**Male copulatory organ**	**Grouping in Gottwald (1983)**
*Parvocythere directocostata*	220	divided	S?	2	sym	D
*Parvocythere dentata*	190–210	present	S	2	sym	D
*Parvocythere fernandinensis*	185–193	present	S	2	sym	E
*Parvocythere galapagoensis*	197–220	present	S	2	sym	D
*Parvocythere schmidti*	168–193	present	S	2	sym	D
*Parvocythere subterranea*	160–181	present	S	2	sym	D
*Parvocythere psammophila*	168	present	S	2	sym	D
*Parvocythere mauiensis*	217	present	S	2	-	-
*Parvocythere japonica*	170–190	present	S	2	sym	D
*Parvocythere marginocostata*	190–240	absent	N	2	sym	E
*Parvocythere dimorpha*	170–180	absent	N	2	asym	D
*Parvocythere hartmanni*	150–160	absent	N	1+1	sym	D
Parvocythere supralitoralis*	126–139	absent	N	1+1	sym	D
*Parvocythere gracilis* sp. n.	156–170	absent	N	1+1	asym	D
*Parvocythere elongata*	150–180	absent	N	1	asym	E
*Parvocythere* spec. A in Gottwald (1983)*	155	absent	N	1	asym	D
*Parvocythere gottwaldi* sp. n.	168–176	absent	N	1	asym	D

**Figure 12.**
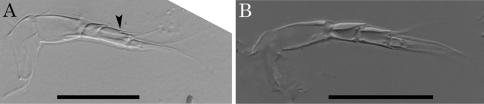
Morphological variations of antennule of *Parvocythere* species. **A** antennule of *Parvocythere japonica* (holotype) **B** antennule of *Parvocythere gottwaldi* sp. n. Arrow indicates suture on the middle of fourth podomere. Scale bars indicate 30 µm, respectively.

Two character states are observed in the antennular fourth podomere of this genus: one is the presence of a suture on the middle of the podomere with two setae on its anterior and posterior sides; the other is the absence of the suture and setae (Fig. 12). As an exception, *Parvocythere directocostata* Hartmann, 1974 has a six-segmented antennula in which the fourth and fifth podomeres correspond to the sutured fourth podomere. The suture on antennular fourth podomere is observed in the following eight species: *Parvocythere dentata*, *Parvocythere fernandinensis* Gottwald, 1983, *Parvocythere galapagoensis*, *Parvocythere schmidti*, *Parvocythere subterranea*, *Parvocythere psammophila* Gottwald, 1983, *Parvocythere mauiensis* and *Parvocythere japonica*. On the other hand, no suture on the antennular fourth podomere is observed in the other eight species: *Parvocythere elongata*, *Parvocythere hartmanni* Marinov, 1962, *Parvocythere dimorpha*, *Parvocythere marginocostata* Hartmann, 1974, *Parvocythere supralitoralis*, *Parvocythere gottwaldi* sp. n., *Parvocythere gracilis* sp. n. and *Parvocythere* spec. A, referred in Gottwald (1983). These two groups are characterised by the presence/absence of suture on the antennular fourth podomere, the former group being called “Group S” and the latter group “Group N” in the present paper. The suture on the antennular fourth podomere is a character observed in the adult individuals of most of cytheroid taxa (see Maddocks 2000), therefore this character can be regarded as a plesiomorphy of the *Parvocythere* species. On the other hand, the lack of the suture is a character observed in juveniles up to the A-4 instar in all the podocopan superfamilies (Smith and Tsukagoshi 2005). This character can be regarded as apomorphy in *Parvocythere*. Moreover this character suggests that the ancestor of Group N had gone through heterochronic (pedomorphic) evolution in its antennula. The unique character of six-segmented antennula in *Parvocythere directocostata* can be understood as “division of segment 4” which is a plesiomorphic character in the podocopids (Smith and Tsukagoshi 2005), but it is hard to decide really whether this character would be the true ancestral state or secondarily occurring one.

**Figure 13.**
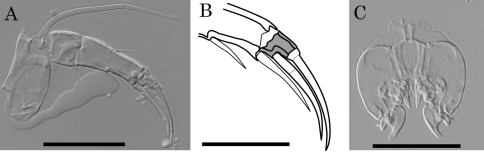
Antenna and male copulatory organ of *Parvocythere japonica* (holotype) as a representative of the Group S. **A** antenna **B** sketch of distal region of antenna **C** male copulatory organ. Shaded podomere is distal fourth podomere. Scale bars indicate 30 µm, 20 µm and 50 µm for **A**, **B** and **C** respectively.

Three states of antennal distal end are observed among the *Parvocythere* species (Gottwald 1983): 1) two claws (Fig. 13A and B); 2) one claw and one seta (Fig. 9B); 3) only one claw (Fig. 4B). Since the two distal claws of the antenna is the typical character in the cytheroids, this character state should be regarded as a plesiomorphy of *Parvocythere*. The other two morphotypes indicate a reduction or lack of one of the two distal claws, and the two states can be regarded as apomorphic characters in this genus. Although all species of the Group S retain the two antennal distal claws, the species of Group N show all three antennal character states: i.e., *Parvocythere dimorpha* and *Parvocythere marginocostata* own two claws; *Parvocythere hartmanni*, *Parvocythere supralitoralis* and *Parvocythere gracilis* sp. n. own a claw and a seta; *Parvocythere elongata*, *Parvocythere gottwaldi* sp. n. and *Parvocythere* spec. A, referred in Gottwald (1983), have only one claw (Table 3). The reductive distal claw therefore appears only in the species of Group N, and the degree of reduction is varied. The interstitial cobanocytherid species also have one claw and one seta on the distal end of the antenna (Hartmann 1959, Schornikov 1975, Gottwald 1983, Higashi and Tsukagoshi 2011). The reductive antennal distal claw is probably a character status related to the adaptation to the interstitial environment.

One of the paired male copulatory organs (hemipenes) is strongly reduced in *Parvocythere elongata*, *Parvocythere dimorpha* and *Parvocythere* spec. A referred in Gottwald (1983), belonging to Group N. Their right hemipenes are reduced in *Parvocythere elongata*, and *Parvocythere* spec. A (Gottwald 1983), but which specific side of the reductive hemipenis was not identified in *Parvocythere dimorpha* (Hartmann 1959, 1974, Gottwald 1983). *Parvocythere gottwaldi* sp. n. and *Parvocythere gracilis* sp. n. also have a reduced right organ (Figs 5 and 10). This character state should be an apomorphy because it cannot be observed in other cytheroids. The reduction of one of the hemipenes, of which the length is approximately one third of body length in *Parvocythere* species, seems to be relevant to the reduction of the body size for the adaptation to the narrow interstices of sediments (Hartmann 1973). On the other hand, all species of the Group S have a symmetric male copulatory organ (Fig. 13C), which can be observed in the almost all podocopans.

Regarding the carapace, the two groups do not show any remarkable character differences. The many species of Group N, however, are relatively small (< 160 µm of the carapace length) (see Table 3). Actually, the carapace length is within the range of 160–220 µm in Group S but of 126–180 µm in Group N, except for the large *Parvocythere marginocostata* (190–240 µm), so most species of Group N have smaller carapaces. The smaller body size can be regarded as an adaptive character to the interstitial life (e.g. Hartmann 1973, Maddocks 1976, Westheide 1987, Giere 2008). Group N thus includes many derived species which could have undergone miniaturisation.

**Figure 14.**
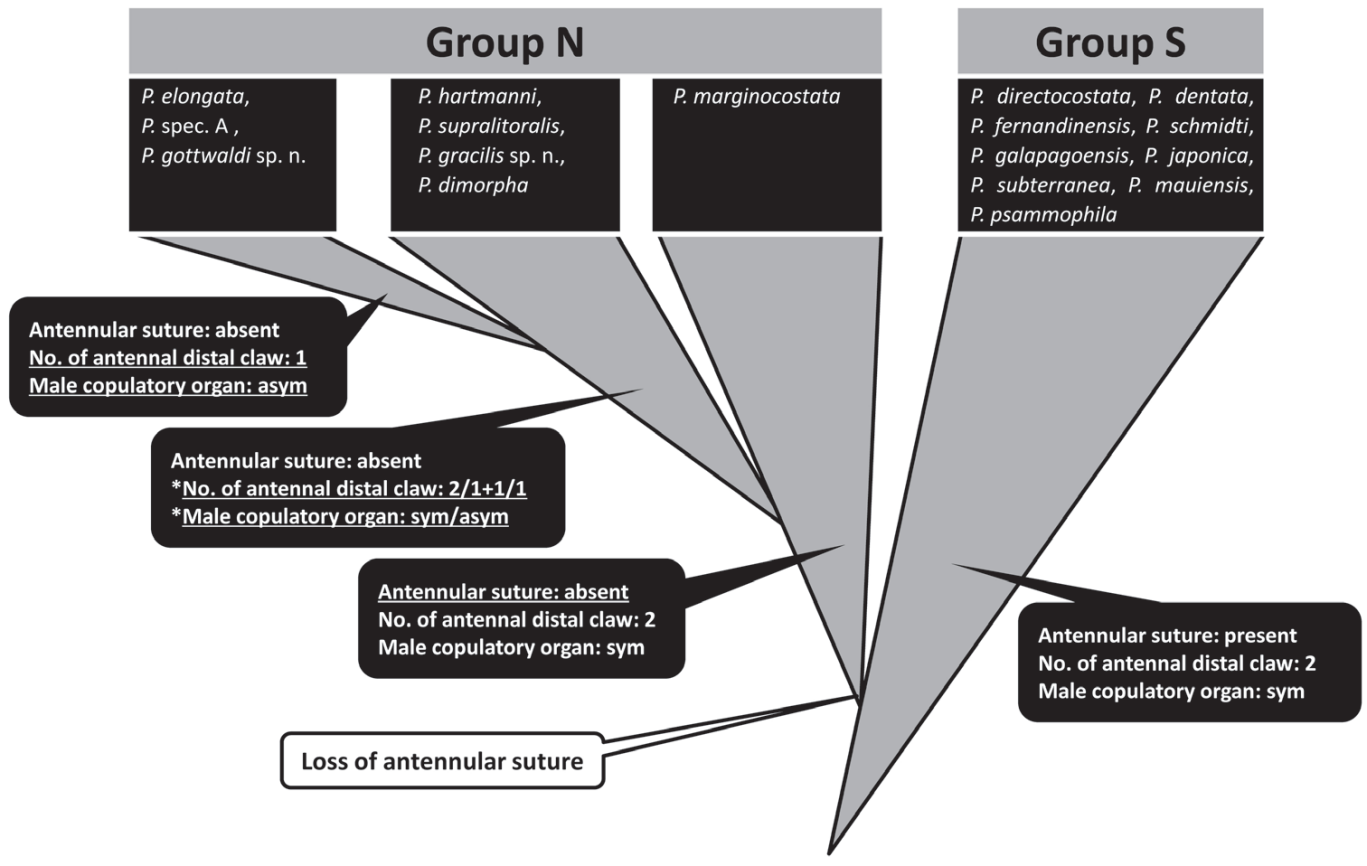
Schematic of inferred evolutionary relationships of the *Parvocythere* species. Triangles coloured in gray indicate each group of species. Species in blackly closed squares indicate them classified into each group. The characters of antennula, antenna and male copulatory organ are represented in the blackly closed balloons and under-lined characters are novel ones. Evolutionary change is represented in an open balloon. The species in the second step of Group N have only one of the reductive states: reductive one of two antennal distal claws or one of pared male copulatory organ (with an asterisk “*”).

The *Parvocythere* species can be divided into Group S, maintaining plesiomorphies, and Group N which includes derived species developing apomorphies as reductive appendages, male copulatory organs, and smaller carapace (Fig. 14). Although Gottwald (1983) classified the *Parvocythere* species into the two groups, i.e. the *elongata* group and the *dentata* group, based only on the morphology of the male copulatory organ, they have no character state shared within each group in their carapace and appendages. On the one hand, the two groups in the present study should reflect the evolutionary tendency of the genus *Parvocythere*. Group S shows many characters regarded as the plesiomorphies of the genus. This does not always provide evidence for the monophyly of the group, but suggests the possibility that the species of Group N were derived after the Group S. On the other hand, the lack of the suture on the middle of antennular fourth podomere is a unique character, and probably a synapomorphy indicating monophyly of Group N. Moreover, the reduction of antennal distal claws and the asymmetric male copulatory organ, which are recognised only in Group N, can also be regarded as apomorphy. Therefore, *Parvocythere marginocostata*, the only species possessing two antennal distal claws, symmetric male copulatory organ and large body size in the Group N, should retain ancestral status in this group. The other species of Group N have some reductive characters, but the precise combination of those characters is not evident: *Parvocythere dimorpha* has the antenna with two distal claw and asymmetric male copulatory organs; *Parvocythere hartmanni* and *Parvocythere supralitoralis* have the antenna with a distal claw and a seta and symmetric male copulatory organ; *Parvocythere gracilis* sp. n. has the antenna with a distal claw and a seta and an asymmetric male copulatory organ (see Table 3). This suggests that homoplastic changes occurred in either the antennal distal claw or the male copulatory organ in Group N. However, the species which bear only one antennal distal claw, i.e. *Parvocythere elongata*, *Parvocythere* spec. A referred in Gottwald (1983) and *Parvocythere gottwaldi* sp. n., have exclusively asymmetric male copulatory organ. These species seem to be closely related to each other (probably monophyly), and are the most derived species in the genus *Parvocythere* (Fig. 14).

The genus *Parvocythere* is composed of only interstitial species and characterised by some reductive characters (e.g. small body size less than 250 μm of the length, only two paired walking legs and the absence of eyes), regarded as the result of an adaptation to the interstitial environment (Hartmann 1959, Hartmann 1973, Gottwald 1983). In the lineage of this exclusively interstitial genus, Group N is regarded to have been derived from a part of Group S. Thus, the apomorphic characters observed in species of Group N (i.e. no suture on the antennular fourth podomere, reduced/lack of one of distal claws on the antenna and asymmetric male copulatory organ) should be formed after this exclusively interstitial genus had been derived from a taxon inhabiting other habitats. In addition to this, the reduction/lack of one antennal distal claw and reduction of one of paired male copulatory organ could be expected to decrease the volume of the animal body. Actually, the species of Group N possessing these characters show a shorter valve length than the species of the Group S (Table 3). Consequently, the apomorphic characters found in the antenna and male copulatory organ of some Group N species can be related to miniaturisation, an adaptive evolutionary trend to the narrow interstitial habitat. The evolutionary process assumed by the morphological variation among the *Parvocythere* species therefore offers the possibility that some adaptive characters of interstitial animals occur after colonisation into the interstitial habitat.

## Conclusion

The morphological comparison among *Parvocythere* species, including the two new species, suggests that Group N derived after Group S through the heterochronic change of antennula, and that the reduction of antennal distal claw and/or the forming of the asymmetric male copulatory organ occurred as an evolutionary tendency within Group N (Fig. 14). These morphological variations among the exclusively interstitial *Parvocythere* species suggest that animal taxa specialised for the interstitial life could also evolve further characters related to their adaptation.

## Supplementary Material

XML Treatment for Parvocythere gottwaldi

XML Treatment for Parvocythere gracilis
